# Supplementary material for: Experimental semi-autonomous eigensolver using reinforcement learning
Source: Sci Rep. 2021 Jun 10;11:12241. doi: 10.1038/s41598-021-90534-7 (PMC8192530; doi:10.1038/s41598-021-90534-7)
Supplement: Supplementary file 1 — Supplementary Information. [file 41598_2021_90534_MOESM1_ESM.pdf]

Experimental semi-autonomous eigensolver using reinforcement learning

C.-Y. Pan, M. Hao, N. Barraza, E. Solano and F. Albarrán-Arriagada

A Derivation of Eq. (17).

Using Eq. (2) and Eq. (3) of the main text, the operator $\hat{\mathscr{O}}\tau$ for a two-level system is write as

$$\hat{\mathscr{O}} = \alpha^{(0)}|\mathscr{E}^{(0)}\rangle\langle\mathscr{E}^{(0)}| + \alpha^{(1)}|\mathscr{E}^{(1)}\rangle\langle\mathscr{E}^{(1)}|, \tag{1}$$

then, the environment operator E given by

$$\hat{E} = e^{-i\hat{\mathscr{O}}\tau} = e^{-i\alpha^{(0)}}|\mathscr{E}^{(0)}\rangle\langle\mathscr{E}^{(0)}| + e^{-i\alpha^{(1)}}|\mathscr{E}^{(1)}\rangle\langle\mathscr{E}^{(1)}|, \tag{2}$$

According to Eq. (14) (main text), the agent state at the end of the protocol (after N iterations) reads

$$|\mathscr{A}_N^{(j)}\rangle = \hat{D}_N|j\rangle, \tag{3}$$

with $j = \{0,1\}$, it means

$$\hat{D}_N = |\mathscr{A}_N^{(0)}\rangle\langle 0| + |\mathscr{A}_N^{(1)}\rangle\langle 1|. \tag{4}$$

Without loss of generality, we suppose that the fidelity $\mathscr{F} = |\langle\mathscr{E}^{(0)}|\mathscr{A}_N^{(0)}\rangle|^2 > |\langle\mathscr{E}^{(0)}|\mathscr{A}_N^{(1)}\rangle|^2$, then

$$|\mathscr{A}_N^{(0)}\rangle = \sqrt{\mathscr{F}}|\mathscr{E}^{(0)}\rangle + e^{i\varphi}\sqrt{1-\mathscr{F}}|\mathscr{E}^{(0)}\rangle, \tag{5}$$

$\varphi \in [0,2\pi]$. As $|\mathscr{A}_N^{(0)}\rangle$ and $|\mathscr{A}_N^{(1)}\rangle$ are orthogonal, we have

$$|\mathscr{A}_N^{(1)}\rangle = \sqrt{1-\mathscr{F}}|\mathscr{E}^{(0)}\rangle - e^{i\varphi}\sqrt{\mathscr{F}}|\mathscr{E}^{(0)}\rangle. \tag{6}$$

Now, the probability to measure the state $|0\rangle$ at the end of the protocol (after N iterations is given by

$$\begin{aligned}
P_0 &= |\langle 0|\hat{D}_N^{\dagger}E\hat{D}_N|0\rangle|^2 \\
&= |\langle 0|\left(|0\rangle\langle\mathscr{A}_N^{(0)}| + |1\rangle\langle\mathscr{A}_N^{(1)}|\right)\left(e^{-i\alpha^{(0)}}|\mathscr{E}^{(0)}\rangle\langle\mathscr{E}^{(0)}| + e^{-i\alpha^{(1)}}|\mathscr{E}^{(1)}\rangle\langle\mathscr{E}^{(1)}|\right)\left(|\mathscr{A}_N^{(0)}\rangle\langle 0| + |\mathscr{A}_N^{(1)}\rangle\langle 1|\right)|0\rangle|^2 \\
&= |\langle\mathscr{A}_N^{(0)}|\left(e^{-i\alpha^{(0)}}|\mathscr{E}^{(0)}\rangle\langle\mathscr{E}^{(0)}| + e^{-i\alpha^{(1)}}|\mathscr{E}^{(1)}\rangle\langle\mathscr{E}^{(1)}|\right)|\mathscr{A}_N^{(0)}\rangle|^2 = |e^{-i\alpha^{(0)}}\mathscr{F} + e^{-i\alpha^{(1)}}(1-\mathscr{F})|^2 \\
&= \left[e^{-i\alpha^{(0)}}\mathscr{F} + e^{-i\alpha^{(1)}}(1-\mathscr{F})\right]\left[e^{i\alpha^{(0)}}\mathscr{F} + e^{i\alpha^{(1)}}(1-\mathscr{F})\right] = \mathscr{F}^2 + \left(e^{i\Delta} + e^{-i\Delta}\right)\mathscr{F}(1-\mathscr{F}) + (1-\mathscr{F})^2 \\
&= \mathscr{F}^2 + [\mathscr{F}^2 - 2\mathscr{F} + 1] + 2\cos(\Delta)\mathscr{F}(1-\mathscr{F}) \\
&\Rightarrow P_0 = 2\mathscr{F}(\mathscr{F}-1)[1-\cos(\Delta)] + 1,
\end{aligned} \tag{7}$$

with $\Delta = |\alpha^{(1)} - \alpha^{(0)}|$, recovering the expression given by Eq. (17) in the main text.

B DATA SETS OF SINGLE-QUBIT CASES

Appendix Table 1. Data set of $\hat{\mathscr{O}}\tau = \frac{\pi}{2}\sigma_x$

Ex[1]	1	2	3	4	5	6	7	8	9	10
N	51	59	52	167	112	205	54	116	57	43
P_0	0.981	0.963	0.884	0.980	0.947	0.990	0.969	0.706	0.895	0.940
$\mathscr{F}$	0.995	0.991	0.970	0.995	0.987	0.997	0.992	0.920	0.973	0.985
Ex	11	12	13	14	15	16	17	18	19	20
N	185	162	107	113	64	64	96	190	42	111
P_0	0.893	0.928	0.782	0.972	0.836	0.917	0.683	0.996	0.983	0.981
$\mathscr{F}$	0.972	0.982	0.942	0.993	0.957	0.978	0.913	0.991	0.996	0.995
Ex	21	22	23	24	25	26	27	28	29	30
N	32	79	61	25	161	86	107	32	28	528
P_0	0.996	0.896	0.977	0.974	0.950	0.913	0.984	0.977	0.982	0.946
$\mathscr{F}$	0.991	0.973	0.994	0.993	0.987	0.978	0.996	0.994	0.995	0.986
Ex	31	32	33	34	35	36	37	38	39	40
N	44	85	94	39	149	25	33	63	197	198
P_0	0.858	0.854	0.919	0.970	0.889	0.930	0.978	0.889	0.936	0.949
$\mathscr{F}$	0.963	0.962	0.979	0.992	0.971	0.982	0.994	0.971	0.984	0.987

Appendix Table 2. Data set of $\hat{\mathscr{O}}\tau = \frac{\pi}{4}\sigma_x$

Ex	1	2	3	4	5	6	7	8	9	10
N	48	55	36	287	28	55	348	572	78	284
P_0	0.930	0.981	0.910	0.850	0.940	0.952	0.820	0.936	0.901	0.960
$\mathscr{F}$	0.964	0.990	0.953	0.918	0.969	0.976	0.900	0.967	0.948	0.980
Ex	11	12	13	14	15	16	17	18	19	20
N	93	45	26	92	34	34	25	37	55	46
P_0	0.941	0.992	0.967	0.950	0.975	0.900	0.936	0.912	0.945	0.920
$\mathscr{F}$	0.970	0.996	0.983	0.974	0.987	0.947	0.967	0.954	0.972	0.958
Ex	21	22	23	24	25	26	27	28	29	30
N	47	65	108	109	74	225	141	153	35	54
P_0	0.850	0.878	0.952	0.987	0.980	0.943	0.990	0.985	0.962	0.953
$\mathscr{F}$	0.918	0.935	0.976	0.993	0.990	0.971	0.995	0.992	0.980	0.976
Ex	31	32	33	34	35	36	37	38	39	40
N	114	152	163	125	112	287	55	185	55	108
P_0	0.963	0.945	0.935	0.960	0.975	0.890	0.982	0.958	0.962	0.972
$\mathscr{F}$	0.981	0.972	0.966	0.979	0.987	0.941	0.991	0.979	0.980	0.986

Appendix Table 3. Data set of $\hat{\mathscr{O}}\tau = \cos\frac{1}{10}\sigma_x + \sin\frac{1}{10}\sigma_y$

Ex	1	2	3	4	5	6	7	8	9	10
N	55	49	26	138	320	95	98	31	287	170
P_0	0.956	0.945	0.98	0.916	0.889	0.951	0.868	0.976	0.989	0.989
$\mathscr{F}$	0.984	0.980	0.993	0.969	0.959	0.982	0.951	0.991	0.996	0.996
Ex	11	12	13	14	15	16	17	18	19	20
N	341	221	156	196	180	255	782	186	496	183
P_0	0.972	0.978	0.956	0.982	0.978	0.923	0.965	0.956	0.854	0.959
$\mathscr{F}$	0.990	0.992	0.984	0.994	0.992	0.972	0.987	0.984	0.945	0.985
Ex	21	22	23	24	25	26	27	28	29	30
N	198	98	191	158	125	186	165	145	155	58
P_0	0.955	0.895	0.994	0.965	0.948	0.856	0.962	0.952	0.966	0.952
$\mathscr{F}$	0.984	0.961	0.998	0.987	0.981	0.946	0.984	0.982	0.988	0.983
Ex	31	32	33	34	35	36	37	38	39	40
N	493	435	156	327	535	254	423	138	75	556
P_0	0.943	0.972	0.944	0.954	0.973	0.946	0.955	0.876	0.963	0.82
$\mathscr{F}$	0.979	0.990	0.978	0.983	0.990	0.980	0.984	0.954	0.987	0.932

C DATA SET OF TWO-QUBIT CASE

In this appendix, we will show the all results of two-qubit case, the first line "EX" means that we had run this case 5 times, the second line "N" is the total iterations for each time, the third line "c_{00}" is the number of error "00" when the input state is $|01\rangle$, the forth line and the fifth line "c'_{00}" and "c_{01}" are the times of error "00" and "01", respectively, when input state is $|10\rangle$. The finial four lines are the fidelities of the eigenstates.

Appendix Table 4. Data set for $\hat{\mathscr{O}}\tau$ given by Eq. (19)

Ex	1	2	3	4	5	6	7	8	9	10
N	306	304	188	253	303	219	197	412	130	410
c_{00}	19	15	10	14	11	12	3	7	2	3
c'_{00}	31	0	6	7	4	1	1	10	5	16
c_{01}	11	3	8	2	1	0	5	4	8	9
$\mathscr{F}_{00}$	0.926	0.915	0.911	0.916	0.929	0.925	0.946	0.931	0.966	0.942
$\mathscr{F}_{01}$	0.911	0.900	0.932	0.954	0.951	0.933	0.94	0.898	0.928	0.978
$\mathscr{F}_{10}$	0.912	0.932	0.925	0.911	0.912	0.989	0.932	0.912	0.96	0.938
$\mathscr{F}_{11}$	0.902	0.912	0.909	0.900	0.913	0.981	0.955	0.885	0.934	0.903

Appendix Table 5. Data set of $\hat{\mathscr{O}}\tau$ given by Eq. (23)

Ex	1	2	3	4	5	6	7	8	9	10
N	75	128	90	86	233	92	149	92	92	73
c_{00}	1	5	2	4	6	4	2	4	4	3
c'_{00}	2	1	4	2	1	1	8	1	1	0
c_{01}	2	4	1	1	1	0	7	0	0	1
$\mathscr{F}_{00}$	0.992	0.998	0.986	0.986	0.992	0.994	0.999	0.964	0.991	0.991
$\mathscr{F}_{01}$	0.996	0.942	0.984	0.997	0.990	0.994	0.947	0.956	0.940	0.988
$\mathscr{F}_{10}$	0.997	0.989	0.969	0.996	0.989	0.989	0.957	0.976	0.918	0.975
$\mathscr{F}_{11}$	0.991	0.943	0.971	0.997	0.988	0.992	0.987	0.988	0.959	0.978

Appendix Table 6. Data set of $\hat{\mathscr{O}}\tau$ given by Eq. (25)

Ex	1	2	3	4	5	6	7	8	9	10
N	2370	1068	1702	1559	1711	1360	2174	431	1129	454
c_{00}	52	13	18	68	29	15	45	4	32	13
c'_{00}	26	20	1	47	5	17	30	3	28	10
c_{01}	23	8	21	37	10	15	25	3	29	13
$\mathscr{F}_{00}$	0.924	0.936	0.943	0.953	0.977	0.915	0.908	0.971	0.911	0.971
$\mathscr{F}_{01}$	0.941	0.982	0.901	0.906	0.928	0.928	0.898	0.975	0.923	0.946
$\mathscr{F}_{10}$	0.961	0.964	0.898	0.926	0.937	0.889	0.912	0.989	0.902	0.910
$\mathscr{F}_{11}$	0.953	0.933	0.886	0.938	0.942	0.929	0.905	0.990	0.968	0.909
